# Biomass, Morphology, and Dynamics of the Fine Root System Across a 3,000-M Elevation Gradient on Mt. Kilimanjaro

**DOI:** 10.3389/fpls.2020.00013

**Published:** 2020-02-04

**Authors:** Natalia Sierra Cornejo, Dietrich Hertel, Joscha N. Becker, Andreas Hemp, Christoph Leuschner

**Affiliations:** ^1^Plant Ecology and Ecosystems Research, Albrecht von Haller Institute for Plant Sciences, University of Goettingen, Goettingen, Germany; ^2^Department of Physical Geography, Georg August University of Goettingen, Goettingen, Germany; ^3^Department of Plant Physiology, Bayreuth University, Bayreuth, Germany

**Keywords:** afroalpine heathland, fine root biomass, fine root production, root economics spectrum, root traits, root:shoot ratio, savannah, tropical montane forest

## Abstract

Fine roots (≤2 mm) consume a large proportion of photosynthates and thus play a key role in the global carbon cycle, but our knowledge about fine root biomass, production, and turnover across environmental gradients is insufficient, especially in tropical ecosystems. Root system studies along elevation transects can produce valuable insights into root trait-environment relationships and may help to explore the evidence for a root economics spectrum (RES) that should represent a trait syndrome with a trade-off between resource acquisitive and conservative root traits. We studied fine root biomass, necromass, production, and mean fine root lifespan (the inverse of fine root turnover) of woody plants in six natural tropical ecosystems (savanna, four tropical mountain forest types, tropical alpine heathland) on the southern slope of Mt. Kilimanjaro (Tanzania) between 900 and 4,500 m a.s.l. Fine root biomass and necromass showed a unimodal pattern along the slope with a peak in the moist upper montane forest (~2,800 m), while fine root production varied little between savanna and upper montane forest to decrease toward the alpine zone. Root:shoot ratio (fine root biomass and production related to aboveground biomass) in the tropical montane forest increased exponentially with elevation, while it decreased with precipitation and soil nitrogen availability (decreasing soil C:N ratio). Mean fine root lifespan was lowest in the ecosystems with pronounced resource limitation (savanna at low elevation, alpine heathland at high elevation) and higher in the moist and cool forest belt (~1,800–3,700 m). The variation in root traits across the elevation gradient fits better with the concept of a multi-dimensional RES, as root tissue density and specific root length showed variable relations to each other, which does not agree with a simple trade-off between acquisitive and conservative root traits. In conclusion, despite large variation in fine root biomass, production, and morphology among the different plant species and ecosystems, a general belowground shift in carbohydrate partitioning is evident from 900 to 4,500 m a.s.l., suggesting that plant growth is increasingly limited by nutrient (probably N) shortage toward higher elevations.

## Introduction

Fine roots (i.e. roots ≤2 mm in diameter) are a small but functionally important component of plant biomass, which controls the uptake of water and nutrients and influences biogeochemical cycles through rapid biomass turnover (Vogt et al., 1996; Gill and Jackson, 2000). The lifespan of fine roots, in particular of the smallest 1^st^ and 2^nd^ order rootlets, is short, in woody plants typically not more than a few months (Eissenstat et al., 2000). Despite representing only a few percent of plant biomass, fine roots have been estimated to consume about a third of annual global net primary production, and they represent a major source of soil carbon through root death and rhizodeposition (Jackson et al., 1997; Jones et al., 2004).

Under contrasting environmental conditions, trees adopt different strategies of resource allocation to the fine root system, which all aim at minimizing the resource investment needed to secure nutrient and water uptake. These strategies vary with the species and functional groups, and in dependence on climatic and edaphic factors such as temperature, precipitation, nutrient availability, soil acidity, and soil bulk density (Cairns et al., 1997; Hertel and Leuschner, 2002; Finér et al., 2011). In correspondence, large variation in fine root system size, mean fine root diameter, branching patterns, and fine root turnover has been found in different forest types, even when the general climate is similar (Leuschner and Hertel, 2003; Finér et al., 2011).

Of the fine root properties, lifespan (i.e. the inverse of biomass turnover in a fine root population) has a particularly large influence on the carbon (C) and nitrogen (N) cycles in the rhizosphere and bulk soil. In the last decades, considerable research effort has been directed to those factors that are assumed to control fine root lifespan in different plant life forms and ecosystem types (Eissenstat et al., 2000; Yanai and Eissenstat, 2002; McCormack and Guo, 2014). Among the factors influencing root lifespan are seasonality, edaphic and climatic factors, and biotic conditions, notably species identity, assimilate availability, root infection by mycorrhiza or pathogens, and competition intensity (Eissenstat et al., 2000; Norby and Jackson, 2000; Eissenstat et al., 2013). Root morphological and chemical traits such as specific root length (SRL) (root length per root biomass), root tissue density (RTD) (root biomass per root volume), root N content, and root diameter may also have an important influence on fine root lifespan (Weemstra et al., 2016). Compared to the major advances in our knowledge about leaf functioning (Reich et al., 1997; Wright et al., 2014), little success has been made until recently in the understanding of variation in the carbon economy of roots from different species, functional types, and biomes (Chen et al., 2013; Roumet et al., 2016).

Advancement in our understanding may happen with the introduction of the concept of a root economics spectrum (RES), which mirrors the leaf economics spectrum (LES). The RES concept postulates that a trade-off between acquisition and conservation of resources exists in fine roots, with covariation of fine root traits at the species and ecosystem level (Kramer-Walter et al., 2016; Weemstra et al., 2016; Li et al., 2019). Plants which follow an acquisitive belowground strategy should build roots with high nutrient and water uptake capacity, which typically is linked to high root N content, small root diameter and high specific root length (SRL) and surface area (SRA), and relatively short lifespan. In contrast, the roots of plants with a conservative belowground strategy should maintain roots with relatively low resource uptake rates, low N content, large root diameters but small surface development (low SRL and SRA), and long lifespan. While several trait correlations have been reported (e.g. a negative relation between root N content and lifespan, and a positive between root diameter and lifespan; McCormack et al., 2012; Reich, 2014), the existing empirical evidence for a one-dimensional RES along the acquisitive-conservative axis is not consistent (Weemstra et al., 2016). While a multi-dimensional RES may be a more appropriate concept (Kramer-Walter et al., 2016), it remains unclear whether a RES, if it exists at the species level, can be extrapolated to the community level, as very few studies have tested this hypothesis in natural ecosystems (Holdaway et al., 2011; Prieto et al., 2015; Kramer-Walter et al., 2016; Li et al., 2019). Further studies across different plant functional types and ecosystems along climatic and edaphic gradients using standardized methods are needed before the more general validity of a RES can be accepted.

Mount Kilimanjaro in tropical eastern Africa represents a unique place to study the change in root traits and root system properties along steep gradients in temperature, precipitation, and nutrient (nitrogen) availability at a regional scale. The mountain hosts a great variety of tropical ecosystems including various mountain forest types, savanna woodland, and alpine scrub vegetation, which are dominated by different plant functional types. The plants in these communities represent contrasting life strategies and are constraint by different environmental factors including drought, nutrient deficiency, and cold as well as disturbance agents such as fire and intensive herbivory. As a consequence, plant-internal allocation strategies and root:shoot ratios differ largely, which should lead to broad variation in rooting patterns and root dynamics across the Mt. Kilimanjaro ecosystem matrix. As far as we know, elevation transect studies on fine root biomass and its dynamics have been conducted in tropical mountains of South America (Ecuador: Röderstein et al., 2005; Graefe et al., 2008; Moser et al., 2011, Peru: Girardin et al., 2013, Bolivia: Hertel and Wesche, 2008) and South-east Asia (Malaysia: Kitayama and Aiba, 2002), but not in the tropical mountains of Africa. So far, no clear over-regional pattern has emerged from these studies, highlighting the need for further research. Studies along elevation transects may also allow predictions about future warming effects on the root system, if the studied environmental matrix allows separating the driving factors.

In the framework of a comprehensive investigation of ecosystem structure and functioning along elevation and land use gradients on Mt. Kilimanjaro (the ‘KiLi Project’ of the German Science Foundation DFG), we studied the fine root dynamics and fine root morphology in six major natural ecosystem types on the southern slope of Mt. Kilimanjaro. Main study aim was to identify patterns of fine root biomass, dynamics, and traits along gradients in elevation and associated environmental factors, and to explore the evidence in support of the existence of a RES. Fine root biomass (FRB) and productivity were related to aboveground biomass (AGB) to obtain a measure of belowground carbon partitioning in the different ecosystems. From existing literature overviews of fine root biomass patterns (Leuschner and Hertel, 2003; Hertel and Leuschner, 2010; Finér et al., 2011), we hypothesized that (i) in the ecosystems with harsher environmental conditions and lower productivity (in particular savanna and afroalpine scrub), FRB is lower and fine root turnover and root:shoot ratios are higher than in the moist montane forest belt, (ii) the FRB : AGB and FRP : AGB ratios increase with elevation due to increasing nutrient (N) limitation; and (iii) the independent and partly opposing trends in temperature, moisture and nutrient availability with elevation together with the turnover of species and functional types lead to great variation in root traits, which do not fit to a one-dimensional RES.

## Methods

### Study Area and Design

The study was carried out within the framework of the KiLi project, a larger interdisciplinary research group (DFG-FOR1246) focused on “Kilimanjaro ecosystems under global change: linking biodiversity, biotic interactions, and biogeochemical ecosystem processes”. It is based on the premise that Mt. Kilimanjaro exhibits a vertical zonation of vegetation belts (Hemp, 2006a), which are addressed by studying representative ecosystem types. Our study sites correspond to the plots established in the joint design of the KiLi project, which were selected in terms of representability for a given ecosystem type. Plots were located in core zones of the vegetation belts in order to avoid ecotones (Peters et al., 2019). The plots are located in northern Tanzania (3°4′33″S, 37°21′12″E) on the southern and south-eastern slopes of the mountain. Due to their exposure to humid air masses advected from the Indian Ocean, the southern slopes of Mt. Kilimanjaro are characterized by higher humidity than the northern ones. We chose the moister side of the mountain, because ecosystem diversity is greater here and the vertical climate gradient is more pronounced. The study covered an elevation distance greater than 3,500 m (871 to 4,550 m a.s.l.), reaching from the colline to the alpine belt. Mean annual temperature ranges from 25°C in the savanna at the foothills to 3°C in the afroalpine zone (Appelhans et al., 2015). Rainfall distribution along the slope is determined by the air masses of the intertropical convergence zone (ITCZ) and the south-easterly trade winds, resulting in a bimodal rainfall distribution with a long rainy season from March to May and a shorter one around November (Hemp, 2006a). Along the slope, mean annual precipitation exhibits a unimodal pattern with minimum values around 620 mm yr^-1^ at the foothills and maximum values around 2,600 mm at 2,200 m a.s.l in the middle montane forest, followed by a decrease to 1,208 mm in the afroalpine heathlands (Hemp, 2006a; Appelhans et al., 2015).

The soils on the Kilimanjaro massif all have a roughly similar age and developed from the same volcanic deposits (Dawson, 1992). In the savanna, vertisols have developed, while at higher elevations, andosols are predominant (Zech et al., 2014). Soil depths in savanna, *Erica* forest and afroalpine belt generally do not exceed 30 cm, while they reach several m in the tropical montane forest belt.

Our investigation focuses on the six main natural ecosystem types present along the elevation gradient (**Table 1**), which were studied with five replicates each. In total we sampled 30 plots of 0.25 ha size each. Detailed information about the vertical vegetation zonation and the main plant species is given in Hemp (2006a), adopting the elevation zone terminology of Körner (2012). Briefly, the foothill zone between 800 and 1,100 m a.s.l. is covered by savanna woodlands, with *Acacia-Commiphora* trees dominating the remaining natural vegetation. The lower montane forest zone between 1,600 and 2,000 m a.s.l. is characterized by *Macaranga kilimandscharica*, *Agauria salicifolia*, and, to a lesser degree, *Ocotea usambarensis*. The middle montane forest (*Ocotea* forest) between 2,100 and 2,800 m a.s.l. is dominated by *Ocotea usambarensis, Ilex mitis, Xymalos monospora*, and the tree fern *Cyathea manniana*, and contains a dense understory layer. The upper montane forest (*Podocarpus* forest) from 2,700 to 3,100 m a.s.l. hosts *Podocarpus latifolius* as the dominant tree species, together with *Hagenia abyssinica* and *Prunus africana*. In the highest forest zone, which reaches up to 3,900 m a.s.l, *Erica* bushlands with some remnants of *Erica trimera* forest are dominant. We refer to these four forest ecosystems as the Mt. Kilimanjaro tropical montane forest. Higher up in the afroalpine zone, which extends to 4,550 m a.s.l., heathlands with dwarf shrubs of *Helichrysum* species together with grasses dominate the landscape. The tropical montane forest and afroalpine plots are located inside Kilimanjaro National Park, while the savanna plots and two lower montane forest plots are outside the protected area.

**Table 1:** Site characteristics of the six community types along the slope on Mt. Kilimanjaro.

	Savanna	Lower montane forest	*Ocotea* forest	*Podocarpus* forest	*Erica* forest	*Helichrysum* heathland
Elevation (m a.s.l.)	(871–1,130)	(1,620–2,040)	(2,120–2,750)	(2,720–2,970)	(3,500–3,910)	(3,880–4,550)
MAT (°C)	23.8 (0.5)	15.2 (0.4)	11.6 (0.4)	9.4 (0.2)	6.2 (0.7)	4.0 (0.4)
MAP (mm)	732 (50)	2,164 (34)	2,409 (80)	2,055 (29)	1,523 (64)	1,295 (34.5)
Soil C:N ratio (g g^−1^)	14.44 (0.90)	14.50 (0.96)	18.80 (0.70)	18.80 (1.00)	19.62 (0.43)	10.49 (1.46)
pH (KCl)	5.38–7.27	4.23–5.30	3.49–4.25	3.83–5.35	4.45–4.54	5.00–5.30
WFPS (%) *	24.65 (11.66)	21.59 (8.37)	41.05 (13.24)	36.08 (11.51)	…	…
AGB (Mg ha^−1^)	7.90 (1.95)	360.12 (88.83)	280.46 (48.83)	366.70 (3.46)	57.63 (6.12)	6.34 (2.04)
Stem density (n ha^−1^)	45 (12)	388 (22)	309 (20)	516 (76)	2,086 (724)	…
Basal area (m^2^ ha^−1^)	0.92 (0.24)	49.50 (6.30)	46.96 (5.26)	58.7 (3.62)	15.76 (2.00)	…
Mean tree height (m)	4.63 (0.26)	17.68 (1.64)	12.04 (0.89)	16.16 (1.25)	5.81 (0.24)	…

Given are means with SE (in brackets).*Soil moisture data was only available for one savanna, and two lower montane, Ocotea and Podocarpus forest plots. Note that AGB refers to tree and shrubs biomass only. MAT: mean annual temperature; MAP, mean annual precipitation; WPFS, water-filled pore space; AGB, aboveground biomass. Climatic data from Appelhans et al. (2015), topographic and stand structure data from Hemp (unpublished data), aboveground biomass data from Ensslin et al. (2015) (DBH ≥ 10 cm; allometric equations from Chave et al., 2005), data for Erica forest stand structure from David Schellenberger-Costa (DBH ≥ 5 cm; allometric equations from Dislich et al., 2009) (unpublished data), soil data for mineral topsoil (0–10 cm) from Becker (unpublished data) and Gütlein et al. (2018).

### Fine Root Biomass Inventory

In each plot, at least 10 soil samples were taken at random positions down to 40 cm depth with a soil corer of 3.5 cm diameter. Twelve of the 30 plots were sampled more intensively; here, 15 samples were taken instead. Samples were stored in plastic bags at 5°C until processing. In the laboratory, samples were washed under running water over a sieve of 200 μm mesh size. All root fragments greater than 1 cm in length and ≤2 mm in diameter were selected and subsequently separated under the stereoscope into biomass (living) and necromass (dead) fractions. As indications of root death, we used the degree of root elasticity, the cohesion of cortex, periderm and stele, and the non-turgidity of the cortex (Leuschner et al., 2001). We further separated herb, grass, and fern roots from tree and shrub roots using the lack of visible suberinization and specific root morphological characteristics as criteria. This was done by comparing the samples with root material taken from the study sites. In the subsequent analysis, we only considered tree and shrub roots, as grass, herb, and fern roots have a very different life cycle. Root fractions were dried at 70°C for 48 h and weighed, and the fine root biomass and necromass expressed in Mg d.m. ha^-^¹ to 40 cm depth. In order to estimate the root necromass of fragments less than 1 cm in length, we followed the method introduced by Van Praag et al. (1988) and modified by Hertel and Leuschner (2002). Six samples per plot were selected, and after extracting larger root fragments (> 1 cm length) as described above; they were spread homogenously on a filter paper (730 cm^2^) subdivided into 36 squares. From six randomly selected squares, root fragments were extracted under the microscope. We then extrapolated the mass of the collected small root fragments to the fine root necromass of the remaining samples that were not included in this more detailed analysis, using linear regression equations between the masses of the small root fragments and the larger dead fine root fraction. In cases where a regression equation could not be applied, a mean ratio of small to large root fractions was used.

### Fine Root Morphological Traits

Morphological traits of living fine roots were investigated prior to drying. Each root sample was scanned using an EPSON perfection V700 scanner (EPSON America Inc.). Specific root length (m g^-^¹), specific root area (cm² g^-^¹), mean root diameter (mm), and root tissue density (g cm^-^³) were calculated from the scans and fine root biomass data using WinRhizo software (Régent Instruments Inc., Québec, Canada). We determined the C and N concentrations of the living fine root fraction with a CN elemental analyzer (Vario EL III, Hanau, Germany). Three samples per plot were analyzed, with each sample consisting of two collected samples in the field that were mixed.

### Fine Root Production and Turnover

We estimated annual fine root production with the ingrowth core technique (Majdi, 1996), which has been used in studies worldwide (e.g. Chen et al., 2004; Hendricks et al., 2006; Handa et al., 2008; Adamek et al., 2011; Kubisch et al., 2017). Measurements in temperate forests have shown that this method tends to provide rather conservative values of fine root production compared with other approaches such as sequential coring and minirhizotrons (e.g. Hertel and Leuschner, 2002; Hendricks et al., 2006; Finér et al., 2011). Ingrowth cores have proven to be useful for studying differences in root production between sites, when root growth is fast as in tropical forests (Vogt et al., 1998), and when a large number of plots is investigated synchronously, as is the case in the KiLi project. With more labor-intensive methods such as sequential coring or mini-rhizotrons, it would have been possible to investigate only a small fraction of the plots, thereby limiting the potential for a comparison of the ecosystems. In September 2014 and February 2015 (dry season) we installed 10 ingrowth cores per plot at random locations in the topsoil down to a depth of 40 cm. After extraction of the soil with a corer of 3.5 cm in diameter, we removed all visible roots by hand and refilled the holes with the original root-free soil. We used a soil core of small diameter to minimize the disturbance produced by the method (Hertel and Leuschner, 2002). We restored the original soil horizon sequence and soil bulk density as good as possible. No mesh was used to avoid barriers for root growth and to retain natural conditions (Hertel et al., 2013; Kubisch et al., 2017) without impeding the access of the soil dwelling fauna. The locations were precisely marked with three plastic sticks to enable the correct insertion angle of the core during resampling. In addition, a PVC tube with exactly the same diameter as the soil corer (3.5 cm) was placed on the top of the soil. Resampling was done after one year. We could not collect data on five plots due to logistic problems. The soil samples were processed in the laboratory as described in the previous section (except for the high-resolution analysis of necromass). Fine root production was calculated as ingrown fine root biomass divided by the length of the time interval between the start of recolonization and harvest (Vogt et al., 1998).

To determine the start of recolonization in the different studied ecosystems, we carried out a side study. We placed four additional ingrowth cores in every plot and resampled each one of the cores during the next four months. Accordingly, recolonization started in the savanna and lower montane forest plots roughly two months after core installation and in the montane *Ocotea* forest, *Podocarpus* forest, *Erica* forest, and the alpine dwarf shrub heathlands after three months. Fine root production values were extrapolated to one year to obtain an estimate of annual fine root production in Mg ha^-1^ yr^-1^. Fine root turnover was calculated at the plot level by dividing annual fine root production by mean standing fine root biomass (Gill and Jackson, 2000). Lifespan was then calculated as the inverse of turnover. We assume a steady state between fine root mortality and productivity (Graefe et al., 2008).

### Statistical Analysis

We applied linear mixed effects models (LME) to determine differences in fine root biomass, necromass, productivity, and morphological traits among the studied ecosystems. We used all data points and designated “plot” as a random effect and “ecosystem” as a fixed effect. The Satterthwaite approximation of degrees of freedom was applied to correct for unbalanced sample numbers. For mean root lifespan and nitrogen content, we used mean values per plot and applied ANOVA. Subsequently, Tukey's HSD post-hoc adjustment for multiple comparisons was used to detect differences between ecosystems types. Linear and nonlinear regression analyses were applied to study in the mountain forest plots the relation a) between fine root biomass, necromass, productivity, lifespan, and root morphological traits with the topographic, climatic, soil, and stand structural characteristics; b) among fine root morphological and chemical traits and root lifespan; and c) between the FRB : AGB and FRP : AGB ratios and elevation, mean annual precipitation (MAP), and soil C:N ratio. We conducted the regression analyses with the plot means. A significance level of *p* < 0.05 was used throughout the analyses. Normality and homoscedasticity of model residuals were tested and in the case of lifespan, FRB : AGB and FRP : AGB ratio, data were log-transformed to meet these criteria. In the linear mixed effects models, we added the value of 1 to all fine root biomass and necromass data before log transformation due to the existence of several zero values. Fine root production was log-transformed. These statistical analyses were conducted with R software (R Core Team, 2013). The mixed effects models were calculated with the lmer function from the ‘lmerTest' package (Kuznetsova et al., 2017). Finally, we carried out a principal components analysis (PCA) to assess the interrelation of the fine root-related variables, stand structure, and soil properties among the different ecosystem types along the slope using CANOCO software, version 5.02 (Biometris, Wageningen, the Netherlands).

## Results

### Plant Community Differences in Fine Root Biomass, Necromass, and Root Productivity Along the Elevational Gradient

**Figure 1:**
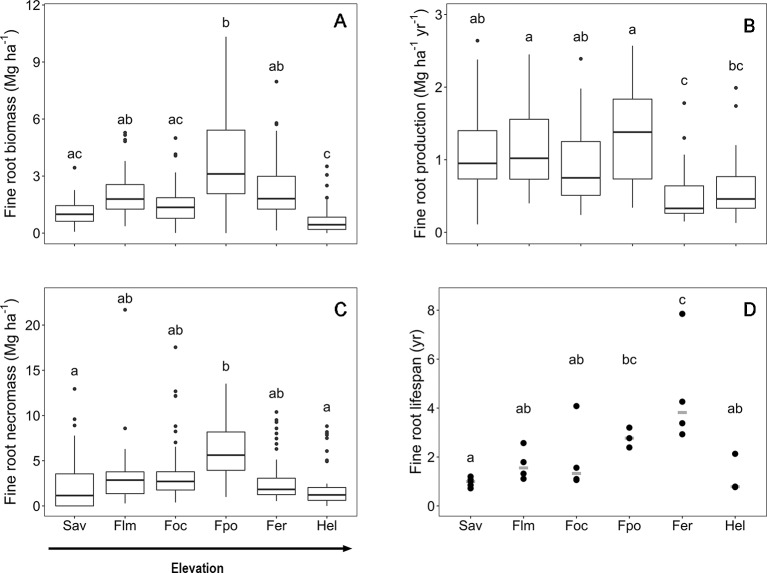
Fine root biomass **(A)**, fine root production **(B)**, fine root necromass **(C)** and mean fine rot lifespan **(D)** in the six communities along the elevation transects on Mt. Kilimanjaro. Fine root biomass and necromass, fine root production and mean fine root lifespan in the six communities along the elevation transects on Mt. Kilimanjaro. Different lower case letters indicate significant differences between communities according to linear mixed effects models with Tukey HSD post-hoc comparison (p < 0.05). Box-whisker plots with median, 25- and 75-percentiles and extremes. Fine root turnover: dots are measurements, the gray line is the median. Sav, savanna; Flm, lower montane forest; Foc, *Ocotea* forest; Fpo, *Podocarpus* forest; Fer, *Erica* forest; Hel, *Helichrysum* heathland.

The fine root biomass and necromass, fine root productivity, and lifespan of woody plants exhibited contrasting elevational patterns along the slope of Mt. Kilimanjaro. Fine root biomass and necromass revealed a unimodal curve with a peak in the *Podocarpus* forest zone (2,720–2,970 m a.s.l.) (**Figure 1**). Fine root production was unaffected by elevation below 3,000 m a.s.l. but decreased higher up the slope toward the *Erica* forest and *Helichrysum* heathland. Furthermore, fine root lifespan linearly increased with elevation toward the tree line, which is formed by *Erica* forest at about 4,000 m a.s.l., followed by a strong decrease toward the alpine *Helichrysum* heathland belt.

Mean fine root biomass and necromass increased threefold from the savanna to the *Podocarpus* forest, where maxima of 3.7 and 6.5 Mg ha^−1^, respectively, were reached (**Table S1**). The subsequent decrease toward the *Helichrysum* heathland led to biomass and necromass minima of 0.8 and 2.3 Mg ha^−1^, respectively. In general, fine root necromass exceeded biomass 1.5- to 3-fold along the slope. Values of carbon content in the aboveground biomass, fine root biomass and in the soil (down to 40 cm depth) are shown in **Table S2**.

Fine root production reached a maximum rate of 1.3 Mg ha^−1^ yr^−1^ in the upper montane *Podocarpus* forest without significant differences to the ecosystems downslope (**Table S1**). The productivity decrease in upslope direction toward the *Erica* forest (0.5 Mg ha^−1^ yr^−1^) was significant.

The mean fine root lifespan of woody plants was lowest in the ecosystems downslope and upslope of the tropical montane forest belt, i.e. the savanna woodland and *Helichrysum* heathland (1.0 and 1.2 yr, respectively) (**Table S1**), which are characterized by relatively harsh environmental conditions. Lifespan was particularly high in the *Erica* forest (4.6 yr) with a 75% higher value than in the savanna.

### Community Differences in Fine Root Morphological and Chemical Traits

**Figure 2:**
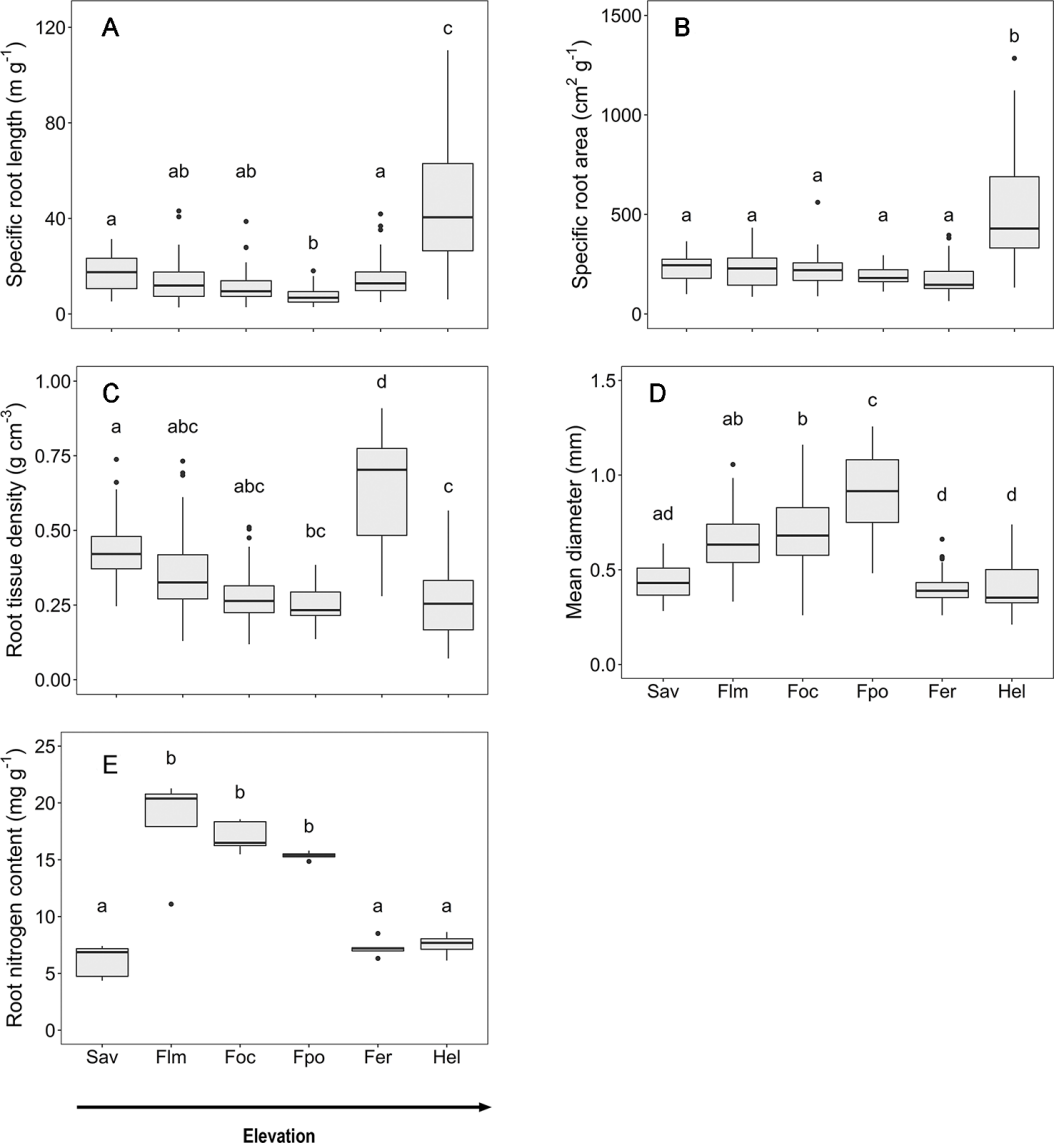
Root morphological and chemical traits for the six communities along the elevation transects on Mt. Kilimanjaro. **(A)** specific root length, **(B)** specific root area, **(C)** root tissue density, **(D)** mean diameter and **(E)** root nitrogen content.. Different lower case letters indicate significant differences between communities according to linear mixed effects models with Tukey HSD post-hoc comparison (p < 0.05) (box-whisker plots with median, 25- and 75-percentiles and extremes). Sav, savanna; Flm, lower montane forest; Foc, *Ocotea* forest; Fpo, *Podocarpus* forest; Fer, *Erica* forest; Hel, *Helichrysum* heathland.

Along the slope, marked changes in the root morphological and chemical traits of the woody plants were observed that are caused by both elevation and community differences in species and functional type composition (**Figure 2**). Mean fine root diameter varied between 0.4 (in the *Erica* forest and *Helichrysum* heathland) and 0.9 mm (in the *Podocarpus* forest). It increased by more than 50% from the savanna to the *Podocarpus* forest, corresponding to minima of SRL and RTD. Community means of SRL and SRA were highest in the *Helichrysum* heathland (47 m g^−1^ and 517 cm^2^ g^−1^, respectively), which is dominated by dwarf shrubs. SRL gradually decreased by almost two third from the savanna woodland (18 m g^−1^) to the upper montane *Podocarpus* forest, which holds the minimum mean value (7 m g^−1^), followed by an increase by 50% toward the *Erica* forest (**Figure 2**). Fine root tissue density (RTD) decreased gradually from the savanna to the *Podocarpus* forest, where it reached its minimum (0.3 g cm^−3^), and increased toward the *Erica* forest again with the maximum (0.7 g cm^−3^), which exceeded the other ecosystems up to 2.5 fold. Root N content reached minima at both high and low elevation (savanna, *Erica* forest, and *Helichrysum* heathland with 6.1, 7.3, and 7.5 mg g^−1^ respectively). It peaked in the lower montane forest (18.3 mg g^−1^) and steadily declined toward the *Erica* forest higher up.

### Relationships Between Fine Root Biomass, Necromass, and Productivity and Elevation, Climate, and Soil in the Montane Forest Belt

**Figure 3:**
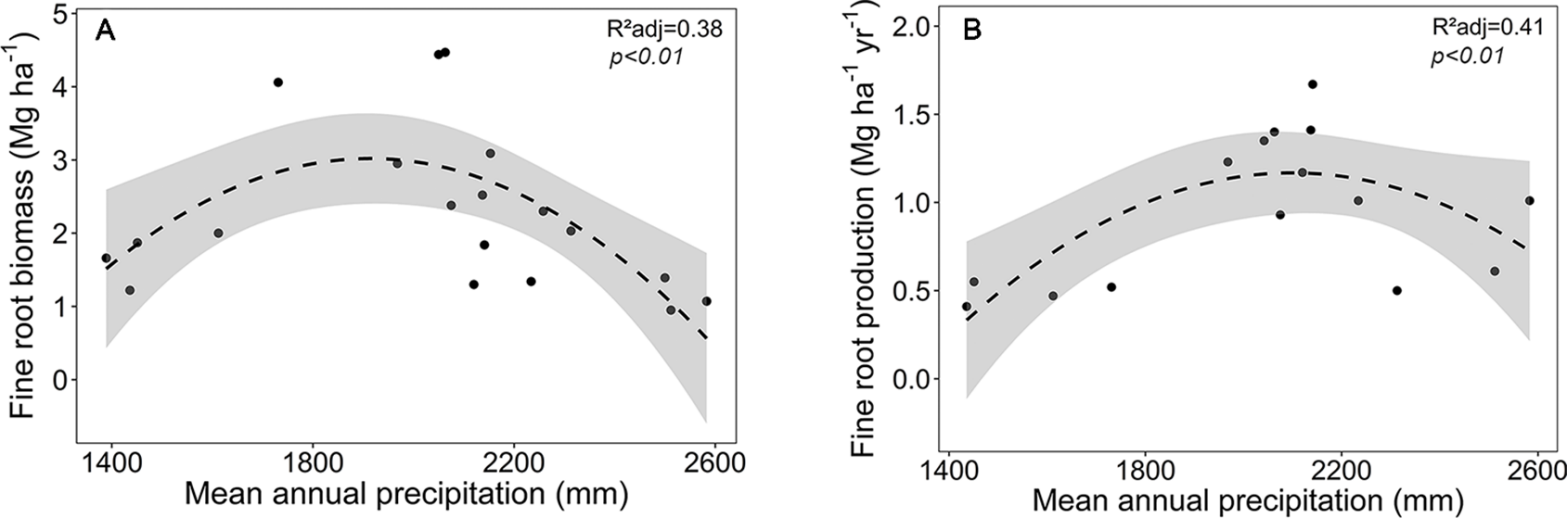
Dependence of **(A)** fine root biomass, and **(B)** fine root production on mean annual precipitation in the four forest communities on Mt. Kilimanjaro (lower montane forest, *Ocotea* forest, *Podocarpus* forest, *Erica* forest). Dashed lines indicate a 2^nd^ order polynomial regression fitted to the data and gray areas display the 95% confidence interval.

**Figure 4:**
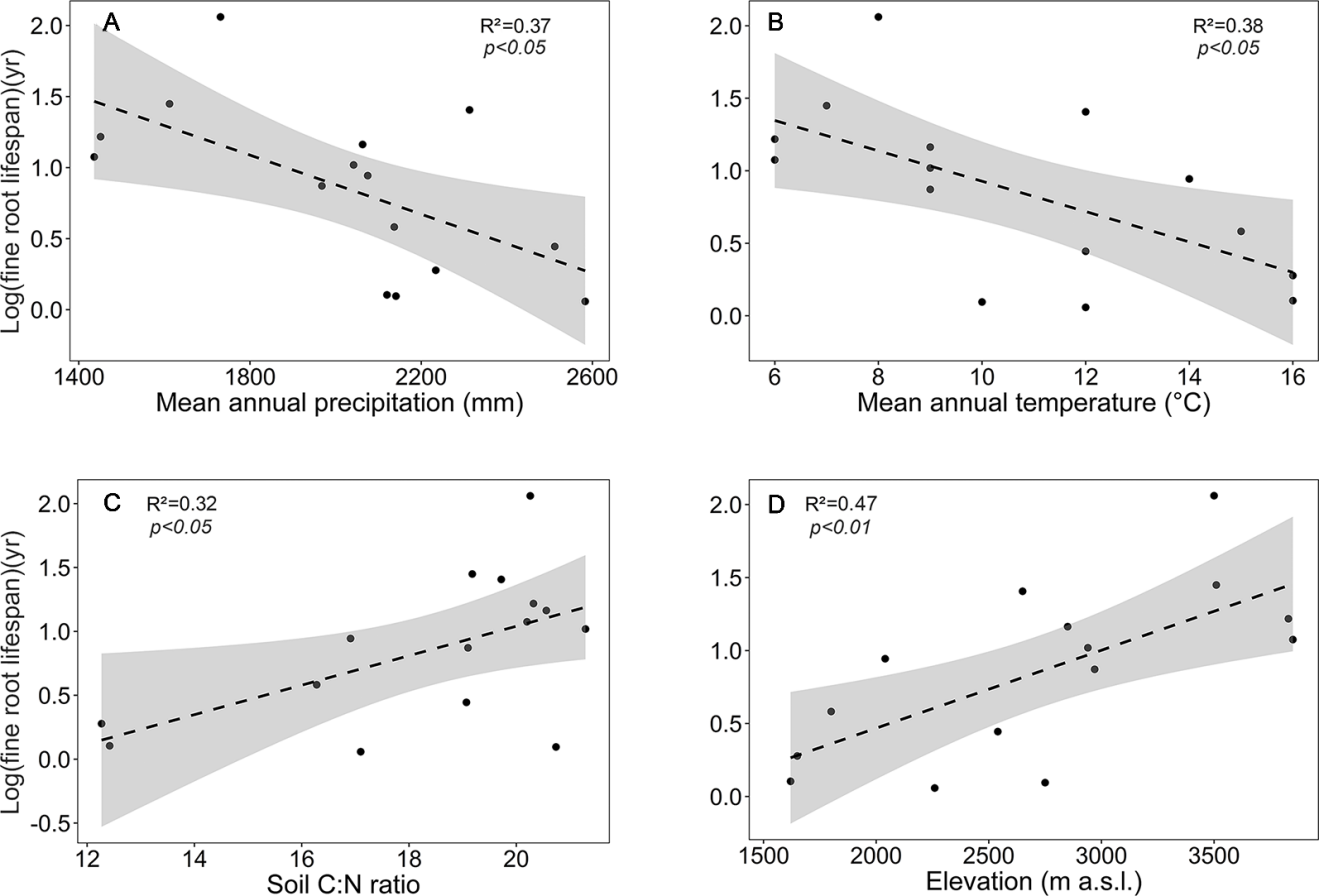
Dependence of log (mean fine root lifespan) on **(A)** mean annual precipitation, **(B)** mean annual temperature, **(C)** soil C:N ratio, and **(D)** elevation in the four forest communities. Dashed lines indicate the linear regression and gray areas the 95% confidence interval.

Focusing on the ecosystems in the montane forest belt, neither fine root biomass or necromass, nor fine root production revealed a significant dependence on elevation (**Table S3**), demonstrating the dominant influence of community composition on these variables. FRB and FRP showed a unimodal relation to mean annual precipitation with a peak at about 2,000 mm (**Figure 3**), but both variables were unrelated to temperature and edaphic parameters (soil C:N and pH). Neither stem density nor aboveground biomass influenced FRB across the different tropical montane forest communities. Stand basal area, but not aboveground biomass, had a positive influence on FRP. Mean fine root lifespan significantly increased with elevation (p < 0.01) and decreased with increasing precipitation and temperature (**Table S3**, **Figure 4**). Positive effects were also found for soil C:N ratio and stem density, while aboveground biomass and basal area correlated negatively with lifespan. The estimates of the simple linear and non-linear regression models among fine root related variables and abiotic and biotic factors are shown in **Table S4**.

**Figure 5:**
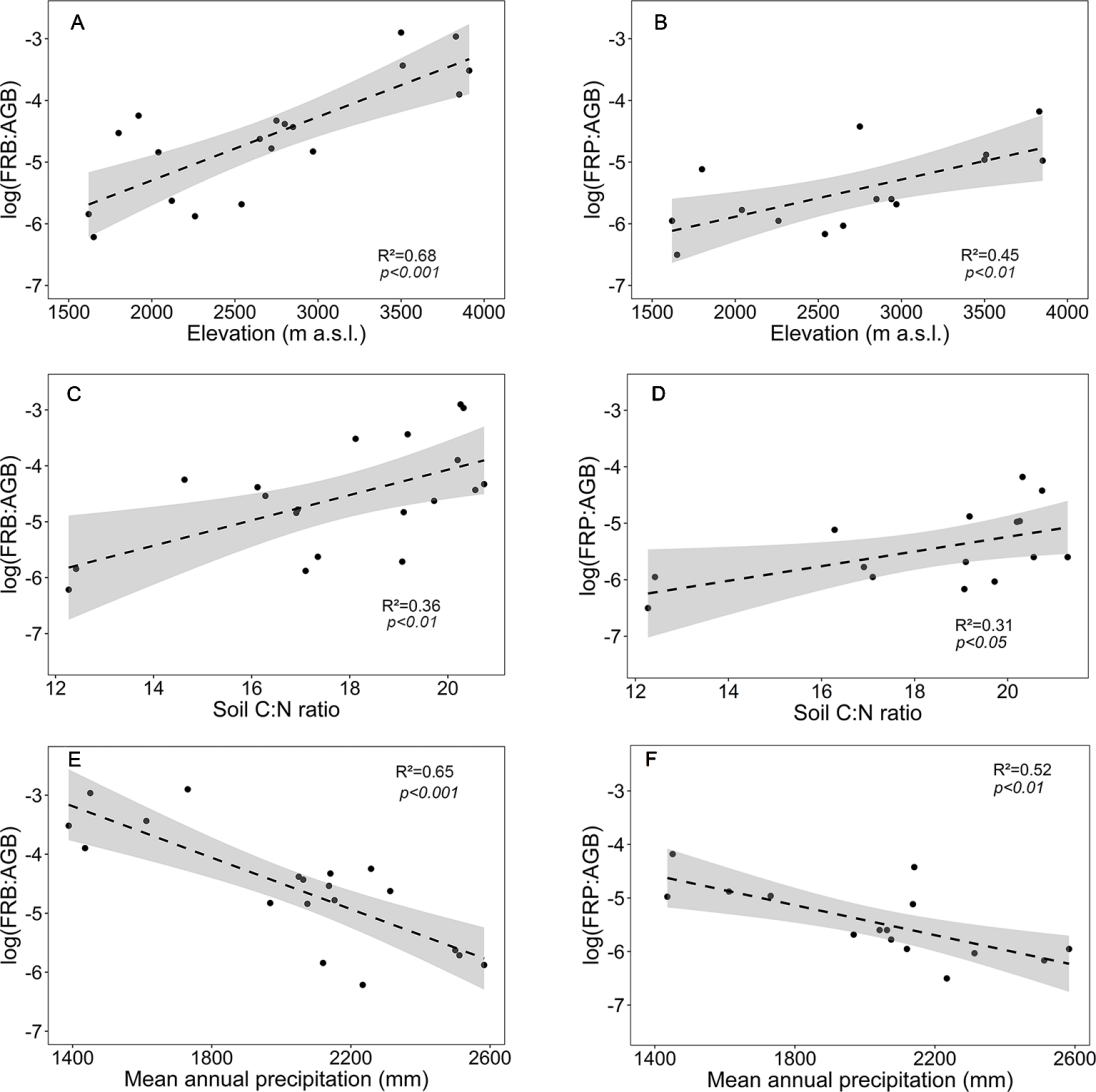
Dependence of fine root biomass to aboveground biomass ratio (log(FRB : AGB)) or fine root production to aboveground biomass ratio (log(FRP : AGB)) on **(A**, **B)** elevation, **(C**, **D)** soil C:N ratio and **(E**, **F)** mean annual precipitation for the four forest communities. Dashed lines indicate the linear regression and gray areas the 95% confidence interval.

In contrast to the absolute values of fine root biomass and production, the ratios FRB and FRP to aboveground biomass (FRB : AGB, FRP : AGB) significantly increased with elevation (**Figures 5A, B**). Both ratios increased with soil C:N ratio and decreased with mean annual precipitation (**Figures 5C–F**).

### Dependence of Fine Root Morphology and Chemistry on Abiotic and Biotic Factors in the Montane Forest Belt

Mean fine root diameter in its dependence on temperature and annual precipitation was best described with an optimum curve, which peaked at about 10°C and 2,100 mm yr^−1^, corresponding to the upper montane *Podocarpus* forest, while SRL and SRA reached their minima roughly at these conditions. Mean fine root diameter increased with precipitation, but showed a hump-shaped relation to temperature. It tended to be higher in communities with larger aboveground biomass and basal area, but decreased with stem density (**Table S5**). SRL was negatively related to aboveground biomass, and SRA peaked at intermediate soil C:N ratios. The relation of SRL and SRA to temperature was generally weak, while SRA was significantly related to precipitation. Root tissue density tended to increase with decreasing precipitation and temperature. Communities with higher aboveground biomass and basal area tended to have lower RTD, while stem density was positively related to RTD (**Table S5**). Root N content was closely negatively associated with soil C:N ratio and decreased with decreases in temperature and precipitation. Communities with higher biomass and basal area had higher root N contents. The estimates of the simple linear and non-linear regression models among fine root morphological and chemical traits and abiotic and biotic factors are shown in **Table S6**. Most of the abiotic and biotic factors were highly correlated (**Table S7**).

**Figure 6:**
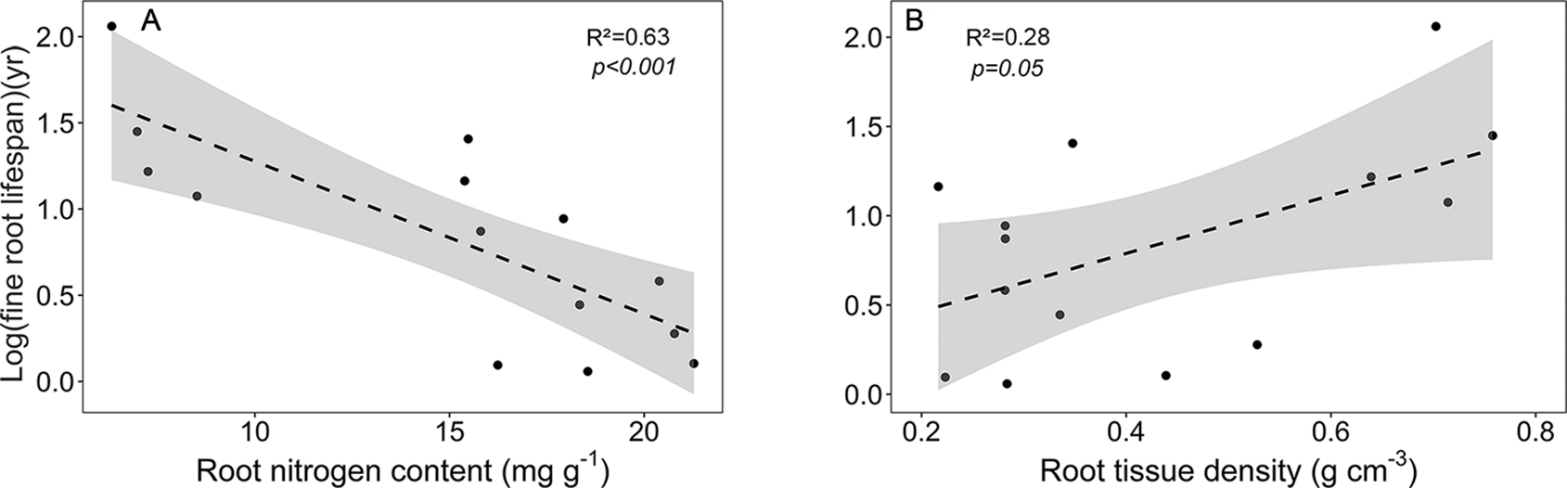
Dependence of log (mean fine root lifespan) on **(A)** root nitrogen content and **(B)** root tissue density. Dashed lines indicate the linear regression and gray areas the 95% confidence interval.

As expected, various root traits were related to each other across our community sample, notably fine root lifespan negatively to root N content, and root tissue density negatively to mean root diameter, SRA, and root N content (**Figure S1**, **Table S8**). The main traits affecting fine root lifespan were root N content (negative relation) and root tissue density (positive relation) (**Figure 6**). FRB increased with mean fine root lifespan, and, unexpectedly, N content increased with mean root diameter (**Table S8**). SRL exhibited a significant relation only to SRA (positive) and to mean root diameter (negative). The estimates of these regression models are given in **Table S9**.

### Principal Components Analysis on the Inter-Relationship Between Belowground, Aboveground and Abiotic Variables

The ordination of the four mountain forest communities by means of principal components analysis (PCA) based on the fine root-related variables, elevation, climatic factors, stand structural characteristics, and soil properties revealed the differentiation of the ecosystems along the elevational gradient (**Figure S2**). Most of the fine root-related variables, elevation, and all of the stand structural characteristics were related to the first axis, while none of the soil properties were associated with it (eigenvalue = 0.66) (**Table 2**). This first axis separated the ecosystems along the elevational gradient, grouping the lower montane forest, *Ocotea* forest, and *Podocarpus* forest together but separating them from the *Erica* forest. The *Erica* forest was associated with high stem density, high FRB : AGB and FRP : AGB ratios, and high root tissue density. The second axis (eigenvalue = 0.22) separated the *Podocarpus* forest from the remaining forest ecosystem types. Fine root biomass, necromass, and soil C:N ratio were positively related to this axis.

**Table 2:** Results of a principal components analysis on the differentiation of the four tropical montane forest ecosystems along the elevation gradient with respect to fine root related variables, elevation, climatic, stand structure, and soil properties.

	**Axis 1 (EV 0.640)**	**Axis 2 (EV 0.221)**	**Axis 3 (EV 0.139)**
*Topographical variable*			
Elevation	**0.856**	0.492	0.158
			
*Climatic factors*			
MAT	**−0.793**	−0.579	−0.190
MAP	**−0.905**	−0.188	0.383
			
*Soil properties*			
C:N soil	0.521	**0.633**	0.572
pH	0.132	0.066	**−0.989**
Soil moisture	−0.249	0.528	**0.812**
			
*Stand structural variables*			
Aboveground biomass	**−0.972**	0.093	−0.216
Stem density	**0.992**	0.064	−0.109
Basal area	**−0.964**	0.250	−0.087
			
*Root related variables*			
Fine root production	**−0.910**	0.316	−0.268
Fine root biomass	−0.125	**0.867**	−0.482
Fine root necromass	−0.368	**0.923**	−0.115
Lifespan	**0.938**	0.339	−0.069
FRB : AGB	**0.992**	0.072	−0.101
FRP : AGB	**0.981**	−0.026	0.190
SRL	0.707	**−0.708**	−0.008
SRA	**−0.775**	−0.505	0.381
RTD	**0.949**	−0.267	−0.167
Mean root diameter	**−0.811**	0.585	−0.009
Root N content	**−0.975**	−0.224	0.008

Given are the loadings of the selected variables along the three explanatory axes. Numbers in brackets indicate the eigenvalues of the axes. Numbers in bold mark the variables with closest correlation to the respective axis.

## Discussion

### Elevational Change in Fine Root Biomass and Productivity

Stand fine root biomass in the soil to 40 cm depth was generally lower in the six studied communities than corresponding values reported from other tropical ecosystems. The mean fine root biomass of woody plants in the savanna woodlands at the foot of Mt. Kilimanjaro (1.0 Mg ha^−1^) was at the lower end of the range of values reported for other tropical savannas and dry forests (0.4 to 11.86 Mg ha^−1^) (Roy and Singh, 1994; Chen et al., 2003; February and Higgins, 2010; Moore et al., 2018). In the present study, we only consider woody roots, whereas most of the cited studies did not differentiate between woody and non-woody roots. Nonetheless, this fact does not explain our low values, as the fraction of non-woody plant roots accounted on average for only 12% of total fine root mass in our savanna plots, the only ecosystem of the study with a significant cover of non-woody plants. More likely causes are the relatively low mean annual precipitation and low aboveground tree biomass at our savanna sites. Furthermore, fine root production in the savanna was also lower and fine root lifespan higher than the range of values given for other savannas and tropical dry forests (ca. 1.0 vs. 2.3 to 14.3 Mg ha^−1^ yr^−1^ and 0.97 yr compared to 0.07 to 0.38 yr, respectively) (Kummerow et al., 1990; Pandey and Singh, 1992; Chen et al., 2004; Moore et al., 2018).

In the tropical montane forest belt, fine root biomass and production (1.5–3.7 Mg ha^−1^ and 0.5–1.3 Mg ha^−1^ yr^−1^) were also in the lower range of values reported in a review of tropical moist forest root data, corresponding closer to values found for tropical lowland forests (0.1–14.4 Mg ha^−1^ and 0.8–21.9 Mg ha^−1^ yr^−1^, respectively, Hertel and Leuschner, 2010). Our values are also smaller than figures reported from a tropical montane forest in Rwanda (7.6 Mg ha^−1^ and 3.8 Mg ha^−1^ yr^−1^, respectively) (Nyirambangutse et al., 2017). Whether this apparent difference is due to species identity effects, or the local climatic and specific edaphic conditions on Mt. Kilimanjaro, must remain open. The use of ingrowth cores, as is done in many other studies, is typically associated with relatively low root fine root production estimates compared to other techniques such as the sequential coring or minirhizotron approaches (Hertel and Leuschner, 2002). This could partly explain the relatively low fine root production values reported in our study. Since all available methods for estimating fine root productivity suffer from specific drawbacks, comparing the results of different studies should be done with great care.

Fine root biomass more than tripled from the savanna at 900 to 1,100 m to the montane *Podocarpus* forest at 2,700 to 2,900 m a.s.l., principally reflecting the large increase in aboveground biomass from the semi-arid foothills to the humid cloud forest belt. Even though the savanna had the lowest fine root biomass of all six ecosystems along the slope, the corresponding FRB : AGB ratio was two orders larger in this ecosystem than in the tropical montane forests higher upslope. The savanna sites at the foot of Mt. Kilimanjaro are characterized not only by seasonal soil moisture deficits, but also by slow N turnover and generally low NH_4_^+^ and NO_3_^-^ concentrations in the soil liquid phase. Grass-dominated patches seem to be exposed to marked N limitation (Becker et al., 2016; Gerschlauer et al., 2016). Nutrient (N) shortage, together with periodic drought, should favor a vegetation with a high FRB : AGB ratio in order to increase nutrient and water uptake and reduce water loss (Kozlowski and Pallardy, 2002; Brunner et al., 2015). In the forest belt (~1,800 to 2,900 m), AGB changed only little with increasing elevation toward the *Podocarpus* forest, while FRB increased from 2.0 to 3.7 Mg ha^−1^. The corresponding increase in the FRB : AGB ratio may be explained by the temperature decrease and its negative effect on nutrient supply. It appears that thermal constraints on soil microbial activity and thus N mineralization rate are fostering carbon investment into the root system at higher elevations on Mt. Kilimanjaro in a similar manner as was found in elevation gradient studies in the Ecuadorian and Peruvian Andes (Moser et al., 2011; Girardin et al., 2013; Leuschner et al., 2013) and other tropical mountain forests (Hertel and Leuschner, 2010).

However, other factors such as moisture seem also to be influential. FRB was somewhat lower in the mid-elevation montane *Ocotea* forest (2,100–2,800 m) than in the other three forest types, which may relate to the favorable growing conditions at this elevation. MAP and soil moisture, leaf litter nutrient concentrations, decomposition rate, and N turnover all were highest in this forest belt (Hemp, 2006a; Appelhans et al., 2015; Becker et al., 2015; Gerschlauer et al., 2016; Becker and Kuzyakov, 2018), suggesting that growth limitation through low N and soil dryness is less important in this than in the other forest communities, while thermal conditions are still relatively favorable. Higher soil fertility is in forests commonly associated with a relatively low fine root biomass (e.g. Vogt et al., 1987; Leuschner and Hertel, 2003; Achat et al., 2008), in accordance with the prediction of resource balance hypothesis (Bloom et al., 1985).

Higher upslope in the upper montane *Podocarpus* and *Erica* forests, N limitation of plant growth is indicated by low leaf litter N contents, and low N:P and high leaf litter C:N ratios (Becker et al., 2015). *Podocarpus* species have been found to form root nodules with symbiotic mycorrhizal fungi (Becking, 1965; Khan, 1967) and also with N_2_-fixing bacteria (Huang et al., 2007), but strong evidence for significant N_2_ fixation in the nodules is lacking so far. Dickie and Holdaway (2010) suggested that nodule formation may be related to a root volume increase in the course of mycorrhizal infection. In the *Erica* forest, the dominant woody species possess specific adaptations to low N availability, notably the capability to access organic N compounds through their ericoid mycorrhiza (Cairney and Meharg, 2003). Limitation by N or other nutrients in the high-elevation vegetation belts on Mt. Kilimanjaro is also indicated by the decrease in fine root N content with elevation. In apparent contradiction, Gütlein et al. (2018) reported relatively high rates of nitrate leaching in the soil of the *Podocarpus* forest. This could result from a relatively slow plant N uptake and uncoupling of supply and plant demand in the cold high-elevation climate.

With the shift of dominant plant life forms from trees to dwarf shrubs at the alpine tree line (~3,900 m a.s.l.), plant strategies to cope with cold and N-poor soils change. The plants in the afroalpine heathland produce on average thinner fine roots with greater surface area development, and fine root biomass per aboveground biomass is much higher. The afroalpine *Helichrysum* heathland with its patchy vegetation structure, low decomposer activity, and slow C and N cycling rates (Gütlein et al., 2017; Becker and Kuzyakov, 2018) may require an extended fine root system to access patches with higher nutrient availability.

### Abiotic and Biotic Drivers of Fine Root Biomass, Productivity, and Fine Root Lifespan

When exploring the effects of elevation, climate, soil, and stand structure on FRB and fine root dynamics for the four montane forest communities while excluding savanna and afroalpine scrub, we find a greater C allocation to the fine root system with increasing elevation and also with factors indicating N shortage, in support of our hypothesis.

At the stand level, the only environmental factor significantly influencing fine root biomass was MAP, displaying a hump-shaped relationship with a peak at about 2,000 mm yr^−1^. This contrasts with root studies along Andean mountain slopes (Moser et al., 2011; Girardin et al., 2013), where FRB increased with elevation and decreasing temperature, while MAP (which was high throughout the transects) was not influential. Studies in tropical, temperate, and boreal forests have found positive, negative, or no relationship between FRB and MAP in single- or multiple-species studies (Joslin et al., 2000; Green et al., 2005; Finér et al., 2007; Hertel et al., 2013). It appears that fine root system size and its relation to precipitation depends largely on species and the range of MAP investigated. It is possible that the optimum curve found for the FRB-MAP relation on Mt. Kilimanjaro is partly caused by species-specific differences in fine root system size, which varies considerably along the slope.

Other than FRB itself, the ratios of fine root biomass and production to aboveground biomass (FRB : AGB and FRP : AGB) were closely negatively related to MAP (p < 0.01). This suggests that rainfall reduction has a larger effect on plant-internal carbohydrate partitioning between root and shoot than on stand fine root biomass itself. However, the FRB : AGB and FRP : AGB ratios also increase with soil C:N ratio and elevation (and decrease with temperature) on Mt. Kilimanjaro, variables partly correlating with MAP, which makes it difficult to disentangle the role of these factors along the slope. Nevertheless, the general picture along the slope of Mt. Kilimanjaro suggests that decreases in temperature, soil moisture, and also nitrogen availability all stimulate higher investment in the fine root system, as is predicted by optimal resource partitioning theory (Bloom et al., 1985). Not only N supply (and that of other nutrients) decreases with declining temperature, but nutrient diffusion in the soil and root N uptake activity as well, which is dependent on ATP and thus plant carbon gain. A fine root study along a 2,000-m elevation transect in the Ecuadorian Andes reached at similar conclusions except for precipitation (Moser et al., 2011).

The significant relations of mean fine root lifespan to elevation and temperature, MAP, and soil C:N ratio across the four forest communities show that lifespan increases under conditions of reduced nutrient and water availability. Under nutrient-poor and cold conditions, plants may be forced to produce root tissue with low nutrient content. Through its sclerenchymatic structure, such tissue is well protected against water loss, herbivory, and pathogen attack. Since resource uptake is typically fairly low, roots can reach a favorable cost/benefit ratio in such resource-limited environments only, when they are long-lived (McComark and Guo, 2014). In accordance, Girardin et al. (2013) observed an increase in fine root lifespan with increasing elevation in the Peruvian Andes, whereas a decrease was reported in the Ecuadorian Andes (Graefe et al., 2008), where temporal soil anoxia occurred as an additional stressor at high elevations (Moser et al., 2011).

It appears that unfavorable growing conditions with pronounced resource limitation can act on root lifespan in two opposing ways, either by increasing or by decreasing longevity. Higher longevity increases the nutrient return on carbon and nutrient investment into root production, while reduced longevity could result in higher uptake rates, when young, physiologically more active fine roots with higher uptake capacity are replacing older, less active roots (Eissenstat et al., 2000). In addition, resource limitation in infertile or cold soils is often associated with physical and/or chemical stress to the root in form of cold damage and toxicity, which may reduce root lifespan through increased mortality, as is the case in the upper montane belt of the Ecuadorian Andes. We explain the increase in fine root lifespan with decreasing resource availability on Mt. Kilimanjaro with the species turnover along the gradients. Thus, more stress-tolerant taxa with a more conservative C economy of their roots replace taxa with more acquisitive, shorter-lived roots, thereby avoiding high root mortality under stress.

The spatial variation in root variables in the Mt. Kilimanjaro environmental matrix is caused by both global climate patterns (e.g. temperature effects on FRB, and FRB : AGB, and FRP : AGB ratios) and local influences (e.g. effects of species, soil, and local topography on fine root lifespan and productivity). Broadly similar fine root patterns as found on Mt. Kilimanjaro might be expected also on other semi-humid African mountains as Mt. Kenya, Mt. Meru, Mt. Elgon and the Aberdare Mountains, which have a similar general climate with comparable vegetation zonation, and join a number of key species (e.g. *Ocotea usambarensis*, *Podocarpus latifolius, Erica* species) (Bussmann, 2006). However, the local conditions likely will cause somewhat deviating rooting patterns in these mountains, as, for example, precipitation levels are relatively high on Mt. Kilimanjaro and a bamboo belt is absent, or, the *Erica* forest of Mt. Kilimanjaro belt is replaced by patchy *Erica* scrub on Mt. Elgon and the Aberdare Mountains (Bussmann, 2006; Hemp, 2006b).

Working along temperature and precipitation gradients might allow some conclusions on the belowground response of these ecosystems to global change. However, a space-for-time approach (Blois et al., 2013) applied to a mountain with a linear temperature, but unimodal precipitation gradient along the slope suggests that predictions about the adaptation of root systems to a warmer and drier future can hardly be made.

### Fine Root Morphology and Chemistry as Dependent on Environment and Species Identity

Our transect covers a broad temperature (25 to 3°C) and precipitation (620 to 2,580 mm) range. The consequently large variation in vegetation types (forest, savanna, alpine scrubland, and heathland) and dominant plant life forms (angiosperm and gymnosperm trees, shrubs, and dwarf shrubs) should be associated with considerable variation in fine root morphology. Indeed, community means of fine root tissue density and root N content varied by a factor of three across the six communities (0.25–0.65 g cm^−3^ and 7–18 mg g^−1^), while the relative constancy of SRL and SRA (with the exception of the higher values in the *Helichrysum* heathland) is remarkable, as it does not reflect the variability in environmental conditions among the ecosystems. The continuous decrease in RTD with increasing elevation up to the *Podocarpus* forest could relate to the increase in moisture availability (Weemstra et al., 2016), which may allow producing less xeromorphic fine roots. The associated increase in mean fine root diameter in upslope direction is best explained by the presence of root nodules in *Podocarpus*, which lack in the other species.

The root morphology change in the lower part of the transect had little influence on root surface development (SRL and SRA), as the root tissue density decrease is roughly compensated by the diameter increase in upslope direction. This suggests that plants reduce their mean fine root diameter toward more xeric, low-elevation sites primarily to increase drought tolerance and, to a lesser extent, for expanding the absorbing surface area and reaching water and nutrient patches (Bardgett et al., 2014). High root tissue density in the savanna and high-elevation *Erica* forest may further be needed to penetrate the hard, drought-affected soil in these communities and to be less susceptible to damage by rhizophagous soil animals (Eissenstat, 1991; Weemstra et al., 2016). A major change in root morphology is encountered in upslope direction from the *Podocarpus* forest to the *Erica* forest, as the dominant woody plants of both communities represent different growth strategies. The high SRL and SRA values of the patchy alpine *Helichrysum* heathland reflect the typically thinner, less suberized, and more tender fine roots of dwarf shrubs, and may help to reach nutrient-rich patches in the generally nutrient-poor soil (Holdaway et al., 2011; Kramer-Walter et al., 2016). In conclusion, elevational changes in water availability, and most likely also N availability, together with species identity effects, seem to be more influential on fine root morphology than the temperature decrease itself.

### Variation in Fine Root Morphological Traits: Is There Evidence in Support of a Root Economics Spectrum (RES)?

Some of our results support the existence of a continuum of fine root trait syndromes along the four tropical montane forest ecosystems, suggesting a trade-off between resource acquisition and conservation. As expected, mean fine root lifespan correlated negatively with root N content and positively with RTD, and SRL (but not SRA) decreased with increasing mean fine root diameter. On the one end of the assumed trait spectrum are species with short-lived roots of low density and high N content and presumably high uptake capacity, on the other end taxa with long-lived, dense, and N-poor roots, which are more resistant to herbivory and abiotic stress, but have lower uptake capacities. However, in our sample, root diameter was negatively related to RTD and positively to N content, i.e. species that produced on average thinner fine roots did this by increasing tissue density, while reducing N content. This is not what is expected from the resource acquisition-conservation trade-off, as thinner fine roots normally have a higher specific surface area, which should, according to the RES concept, be associated with a higher uptake activity (i.e. higher N content) and shorter lifespan (i.e. lower tissue density) to maximize resource acquisition per investment. Therefore, the conventional concept of a RES along an acquisitive-conservative continuum does not fit well to our results.

The recently proposed concept of fine root trait multi-dimensionality has opened new horizons in the study of RES (Kramer-Walter et al., 2016; Valverde-Barrantes and Blackwood, 2016). This concept that was tested in tree seedlings along a soil fertility gradient (Kramer-Walter et al., 2016), states that, while there are root traits associated along one axis in a root economics spectrum (especially RTD), other traits such as diameter and SRL can be associated to the RES in an orthogonal way (Kramer-Walter et al., 2016). Such a multi-dimensionality of the trait space entails that fine root morphology is less constrained than the construction of leaves, enabling different combinations of SRL and RTD within the root economics spectrum. In nutrient-poor soils, plants could either develop roots of high SRL to explore distant nutrient patches, or operate roots with low SRL and long lifespan to reduce C investment in the fine root system. Our results from the Mt. Kilimanjaro environmental matrix seem to support a multi-dimensional RES, as SRL and RTD, contrary to the negative relation assumed in a one-dimensional RES, show indeed variable combinations. The large diversity in species and plant life forms in our ecosystems has also to be taken into account, as root morphology is strongly influenced by genotype, despite great plasticity in fine root traits (Hodge, 2004). This is visible when comparing the *Podocarpus* and *Erica* forests in our study, which both seem to be N-limited, while exhibiting different RTD values as seen in the PCA.

While our results are in line with a multi-dimensional RES at the community level, they do not confirm the assumption of RTD being the most decisive fine root trait along soil fertility gradients (Holdaway et al., 2011; Kramer-Walker et al., 2016). Phylogeny seems to overrule any dependency of RTD on soil fertility, as the conifer *Podocarpus* with root nodules pursues a largely different nutrient acquisition strategy than *Erica* with its ericoid mycorrhiza. Our data further suggest that even some of the more widely accepted root trait correlations may not be universally valid. For example, there is evidence that root lifespan can also increase, and not decrease, with a higher root N content (Gordon and Jackson, 2000; Hendricks et al., 2006).

## Conclusions

To our knowledge, this is the first study on fine root system properties (morphology, biomass, and dynamics) in their dependence on elevation and associated environmental factors in African tropical forests. It complements a few earlier studies in tropical mountains of South America and South-east Asia, but differs from these in that the mountain base is semi-arid and that it extends beyond the alpine tree line. The study covers an altitudinal distance of ca. 3,000 m, which is associated with a turnover in ecosystem types and plant life forms, ranging from low-elevation savannas to afroalpine scrub. This results in remarkably broad fine root trait spectra along the slope. Even when analyzing only the montane forest belt, where differences in vegetation structure are less pronounced, the variation in fine root biomass, productivity, and fine root morphology was still large. This demonstrates that, beside elevation effects, species identity, and phylogeny are playing an important role as factors controlling fine root system properties. The interplay between biotic and several abiotic drivers made it difficult to clearly disentangle the influence of temperature, moisture, and nutrient availability on root properties in the Mt. Kilimanjaro environmental matrix. This is an unavoidable disadvantage of comparative studies along elevation and climatic gradients. Nevertheless, the study revealed important changes in plant carbon allocation patterns along the slope. When expressed in relation to aboveground biomass, FRB and FRP showed close relations to elevation (temperature), MAP, and soil C:N ratio, indicating a general shift in carbohydrate allocation from shoot to root with decreasing resource availability and temperatures. This phenomenon seems to be widespread in tropical as well as extra-tropical regions (e.g. Leuschner et al., 2007; Hertel et al., 2008; Hertel and Leuschner, 2010; Hertel and Schöling, 2011), suggesting a significant role of nutrient availability in the elevational decrease of forest productivity in mountains around the globe.

The analysis of inter-relationships between important root morphological and chemical traits and mean root lifespan revealed several relations consistent with a root economics spectrum, but our data fit better with the concept of multi-dimensionality in the root trait spectrum, and they underpin that root responses to soil fertility are largely dependent on phylogeny and specific root symbionts. Moreover, the majority of species combine acquisitive and conservative root traits in a certain way, and the extremes of the trait spectrum are linked by a continuum of intermediate strategy types. Future studies on a RES and the dependence of root traits on the environment should use standardized root analysis methods, focus more on traits with closer link to physiology (e.g. root respiration, nutrient uptake capacity, root drought and cold resistance), address the effects of biotic interactions, and quantify linkages between aboveground and belowground traits for the dominant species.

## Data Availability Statement

The datasets generated for this study are available on request to the corresponding author.

## Author Contributions

DH developed the study design. NS conducted the field work, data processing and analysis. JB and AH contributed with soil and stand structure data. Data interpretation and paper writing were done by NS, DH and CL with contributions of all authors.

## Funding

This work was supported by the German Research Foundation (Deutsche Forschungsgemeinschaft DFG) within the Research-Unit 1246 (KiLi) which is gratefully acknowledged. We also acknowledge the support by the Open Access Publication Fund of the University of Göttingen.

## Conflict of Interest

The authors declare that the research was conducted in the absence of any commercial or ﬁnancial relationships that could be construed as a potential conﬂict of interest.

## Abbreviations

FRB, fine root biomass; FRN, fine root necromass; FRP, fine root production; RES, root economics spectrum.
