# Supplementary material for: Biomass, Morphology, and Dynamics of the Fine Root System Across a 3,000-M Elevation Gradient on Mt. Kilimanjaro
Source: Front Plant Sci. 2020 Feb 4;11:13. doi: 10.3389/fpls.2020.00013 (PMC7010809; doi:10.3389/fpls.2020.00013)
Supplement: Supplementary file 1 [file Table_1.pdf]

Supplementary material

Table S1. Community means of fine root biomass (FRB), necromass (FRN), productivity (FRP), turnover and mean fine root lifespan, and ratios of FRB to aboveground biomass, FRP to aboveground biomass, and FRB to FRN in the six communities on Mt. Kilimanjaro (means and SE in parentheses).

	Fine root biomass (Mg ha^{-1})	Fine root necromass (Mg ha^{-1})	Fine root production (Mg ha^{-1} yr^{-1})	Fine root turnover (yr^{-1})	Fine root lifespan (yr)	FRB:AGB	FRP:AGB	FRB:FRN
Savanna	1.04 (0.06)	2.47 (0.47)	1.10 (0.08)	1.07 (0.10)	0.97 (0.08)	0.2075 (0.0856)	0.2365 (0.1057)	0.42 (0.11)
Lower montane forest	1.97 (0.27)	2.78 (0.88)	1.13 (0.11)	0.65 (0.11)	1.70 (0.32)	0.0076 (0.0023)	0.0033 (0.0010)	0.71 (0.27)
Ocotea forest	1.46 (0.21)	3.40 (1.04)	0.95 (0.27)	0.68 (0.16)	1.95 (0.72)	0.0065 (0.0021)	0.0048 (0.0024)	0.43 (0.12)
Podocarpus forest	3.74 (0.41)	6.51 (0.59)	1.33 (0.05)	0.36 (0.03)	2.78 (0.23)	0.0102 (0.0012)	0.0036 (0.0001)	0.57 (0.11)
Erica forest	2.16 (0.49)	2.90 (0.77)	0.49 (0.03)	0.25 (0.05)	4.60 (1.12)	0.0377 (0.0067)	0.0092 (0.0020)	0.74 (0.45)
Helichrysum heathland	0.80 (0.34)	2.30 (1.09)	0.69 (0.17)	1.01 (0.27)	1.23 (0.45)	0.1429 (0.0366)	0.1687 (0.0896)	0.35 (0.29)

Table S2. Carbon stocks in aboveground biomass, fine root biomass and soil (until 40 cm depth) in the six communities on Mt. Kilimanjaro (means and SE in parentheses).

	*Aboveground biomass (Mg C ha^{-1})	Fine root biomass (Mg C ha^{-1})	Soil organic C (Mg C ha^{-1})
Savanna	3.81 (0.94)	0.49 (0.03)	109.65 (17.65)
Lower montane forest	173.58 (42.82)	0.90 (0.13)	241.22 (45.74)
Ocotea forest	135.18 (23.54)	0.70 (0.11)	278.96 (16.05)
Podocarpus forest	176.75 (1.67)	1.63 (0.16)	295.55 (16.12)
Erica forest	27.78 (2.95)	1.04 (0.24)	293.70 (5.87)
Helichrysum heathland	3.06 (0.98)	0.35 (0.15)	110.74 (52.86)

*Data on aboveground biomass C from Ensslin and others 2015 (DBH ≥ 10 cm) and Schellenberger-Costa unpublished (*Erica* forest DBH ≥ 5 cm) and data on soil organic C from Becker N. J. unpublished

Table S3. Regression analyses relating fine root biomass, fine root necromass, fine root production and turnover to elevation, climatic, edaphic and stand structural variables.

	Elevation			Mean annual precipitation			Mean annual temperature			Soil C:N ratio	
	r^2adj r^2	*P*		r^2adj r^2	*P*		r^2adj r^2	*P*		r^2adj r^2	*P*
Fine root biomass	0.12	n.s.	*	**0.38**	**<0.01**	*	0.07	n.s.	*	0.04	n.s.
Fine root necromass	0.17	<0.1	*****	0.06	n.s.	*	0.11	n.s.	*	0.09	n.s.
Fine root production	0.25	<0.1	*	**0.41**	**<0.05**	*	0.14	n.s.	-	0.02	n.s.
Log (fine root lifespan)	**0.47**	**<0.01**	**-**	**0.37**	**<0.05**	**-**	**0.38**	**<0.05**		**0.32**	**<0.05**

		pH (KCl)			Aboveground biomass			Stem density			Basal area	
		r^2	*P*		r^2	*P*		r^2	*P*		r^2	*P*
Fine root biomass		0.02	n.s.	-	$3.6\ 10^{-5}$	n.s.		0.08	n.s.		0.03	n.s.
Fine root necromass	-	0.01	n.s.	-	0.01	n.s.	-	0.09	n.s.		0.01	n.s.
Fine root production	-	0.03	n.s.		0.23	<0.1	-	0.24	<0.1		**0.28**	**<0.05**
Log (fine root lifespan)	-	0.05	n.s.	**-**	**0.33**	**<0.05**		**0.52**	**<0.01**	-	0.19	n.s.

Fine root biomass in Mg ha^{-1}, fine root necromass in Mg ha^{-1}, fine root production in Mg ha^{-1} yr^{-1}, mean fine root lifespan in yr, elevation in m a.s.l., mean annual temperature in °C, mean annual precipitation in mm, aboveground biomass in Mg ha^{-1}, stem density in n ha^{-1} and basal area in m² ha^{-1}. Significant relations are marked in bold ($P<0.05$), nonlinear relations are indicated by (*) and negative relations by (-).

Table S4. Estimates of the simple linear and non-linear regression models of fine root biomass and dynamics with the abiotic and biotic factors along the tropical montane forest at Mt. Kilimanjaro.

Fine root variable	Factor	Intercept	Estimate (X)	Estimate (X^2)	P- value
Fine root biomass	Elevation	-5.26	5.55 10^{-3}	-9.54 10^{-7}	n.s.
	MAP	-17.03	2.10 10^{-2}	-5.48 10^{-6}	< 0.01
	MAT	-0.42	0.64	-3.32 10^{-2}	n.s.
	Soil C:N ratio	0.70	0.09		n.s.
	pH (KCL)	0.93	0.31		n.s.
	AGB	2.27	-3.91 10^{-5}		n.s.
	Stem density	2.53	-6.76 10^{-4}		n.s.
	Basal area	2.31	-1.22 10^{-3}		n.s.
Fine root necromass	Elevation	-13.39	1.27 10^{-2}	-2.18 10^{-6}	< 0.1
	MAP	-21.15	2.70 10^{-2}	-7.02 10^{-6}	n.s.
	MAT	-1.83	1.37	-7.20 10^{-2}	n.s.
	Soil C:N ratio	-0.91	0.26		n.s.
	pH (KCL)	2.12	0.31		n.s.
	AGB	4.12	-1.38 10^{-3}		n.s.
	Stem density	5.27	-3.68 10^{-3}		n.s.
	Basal area	3.81	-1.16 10^{-3}		n.s.
	Elevation	1.13 10^{-1}	1.16 10^{-3}	-2.64 10^{-7}	< 0.1
Fine root production	MAP	-7.20	7.97 10^{-3}	-1.90 10^{-6}	<0.05
	MAT	0.47	4.47 10^{-2}		n.s.
	Soil C:N ratio	1.34	-2.14 10^{-2}		n.s.
	pH	1.87	-0.22		n.s.
	AGB	0.65	1.13 10^{-3}		< 0.1
	Stem density	1.11	-1.69 10^{-4}		< 0.1
	Basal area	0.48	1.13 10^{-2}		< 0.05
Log (fine root lifespan)	Elevation	-0.60	5.34 10^{-4}		< 0.01
	MAP	2.96	-1.04 10^{-3}		< 0.05
	MAT	1.97	-0.11		< 0.05
	Soil C:N ratio	-1.27	0.12		< 0.05
	pH (KCL)	2.62	-0.38		n.s.
	AGB	1.36	-1.90 10^{-3}		< 0.05
	Stem density	0.53	3.48 10^{-4}		< 0.01
	Basal area	1.39	-1.29 10^{-2}		n.s.

Table S5. Regression analyses relating five root morphological and chemical traits to elevation and climatic, edaphic and stand structural variables.

		Elevation				Mean annual precipitation				Mean annual temperature				Soil C:N ratio		
		r^2_{adj}/r^2	*P*			*r^2_{adj}/r^2*	*P*			*r^2_{adj}/r^2*	*P*			*r^2_{adj}/r^2*	*P*	
SRL	-	0.15	<0.1	*	**-**	**0.32**	**<0.05**	*****	-	0.14	n.s.	*		0.03	n.s.	*
SRA		0.09	n.s.		-	0.12	n.s.	*****		0.04	ns.s			**0.39**	**<0.01**	*****
RTD	**-**	**0.55**	**<0.001**	*****	**-**	**0.48**	**<0.01**		**-**	**0.28**	**<0.05**	*	-	0.09	n.s.	*
Mean root diameter		**0.43**	**<0.01**	*****		**0.44**	**<0.01**	*****		**0.24**	**<0.05**	*	-	0.005	n.s.	
Root nitrogen content	**-**	**0.74**	**<0.001**			**0.62**	**<0.001**	*****		**0.66**	**<0.001**		**-**	**0.30**	**<0.05**	

		pH (KCl)				Aboveground biomass				Stem density				Basal area	
		r^2_{adj}/r^2	*P*			*r^2_{adj}/r^2*	*P*			*r^2_{adj}/r^2*	*P*			*r^2_{adj}/r^2*	*P*
SRL		0.03	n.s.	*	**-**	**0.21**	**<0.05**			0.03	n.s.	*	-	0.18	<0.1
SRA	-	0.15	n.s.			0.04	n.s.	*	-	0.18	<0.1			0.05	n.s.
RTD		0.18	n.s.	*****	**-**	**0.65**	**<0.001**	*****		**0.52**	**<0.001**		**-**	**0.50**	**<0.001**
Mean root diameter	-	0.21	n.s.	*****		**0.45**	**<0.01**	*****	**-**	**0.35**	**<0.05**	*****		**0.40**	**<0.01**
Root N content	-	0.16	n.s.	*****		**0.72**	**<0.001**	*****	**-**	**0.62**	**<0.001**	*****		**0.63**	**<0.001**

SRL= specific root length in m g^{-1}, SRA= specific root area in cm² g^{-1}, RTD= root tissue density in g cm^{-3}, mean diameter in mm, root nitrogen content in mg g^{-1}, elevation in m a.s.l., mean annual temperature in °C, mean annual precipitation in mm, aboveground biomass in Mg ha^{-1}, stem density in n ha^{-1} and basal area in m² ha^{-1}. Significant relations are marked in bold (P<0.05), 2nd order polynomial relations are indicated by (*) and negative relations by (-).

Table S6. Estimates of the simple linear and non-linear regression models of fine root morphological and chemical traits with the abiotic and biotic factors along the tropical montane forest at Mt. Kilimanjaro.

Fine root variable	Factor	Intercept	Estimate (X)	Estimate (X^2)	P- value
Specific root length	Elevation	41.06	-2.386 10^{-2}	4.522 10^{-6}	< 0.1
	MAP	100.3	-8.94 10^{-2}	2.196e-05	< 0.05
	MAT	32.11	-3.95	0.17539	n.s.
	Soil C:N ratio	-61.96	9.13	-0.2741	n.s.
	pH (KCL)	-113.09	57.52	-6.503	n.s.
	AGB	15.53	-0.01		< 0.05
	Stem density	8.58	7.87 10^{-3}	-1.702e-06	n.s.
	Basal area	16.69	-0.11		< 0.1
Specific root area	Elevation	250.91	-0.02		n.s.
	MAP	521.5	-3.98 10^{-1}	1.143e-04	n.s.
	MAT	170.25	2.75		n.s.
	Soil C:N ratio	-1064.32	157.62	-4.772	< 0.01
	pH	385.03	-42.00		n.s.
	AGB	168.5	3.18 10^{-1}	-5.453e-04	n.s.
	Stem density	215.54	-0.02		< 0.1
	Basal area	174.96	0.60		n.s.
Root tissue density	Elevation	1.47	-9.76 10^{-4}	1.99 10^{-7}	< 0.001
	MAP	1.11	-3.51 10^{-4}		< 0.01
	MAT	1.24	-0.15	6.25 10^{-3}	< 0.05
	Soil C:N ratio	3.16	-0.35	0.01	n.s.
	pH (KCL)	-5.78	2.67	-0.28	n.s.
	AGB	7.45 10^{-1}	-2.66 10^{-3}	3.63 10^{-6}	< 0.001
	Stem density	2.94 10^{-1}	1.22 10^{-4}		< 0.001
	Basal area	0.68	-6.94 10^{-3}		< 0.001
Mean root diameter	Elevation	-8.54 10^{-1}	1.27 10^{-3}	-2.47 10^{-7}	< 0.01
	MAP	-3.02	3.57 10^{-3}	-8.44 10^{-7}	< 0.01
	MAT	-0.35	0.19	-8.27 10^{-3}	< 0.05
	Soil C:N ratio	0.75	-5.69 10^{-3}		n.s.
	pH (KCL)	8.13	-3.33	0.37	n.s.
	AGB	3.01 10^{-1}	2.44 10^{-3}	-3.06 10^{-6}	<0.01
	Stem density	8.69 10^{-1}	-4.36 10^{-4}	7.65 10^{-8}	<0.05
	Basal area				<0.01
Root N content	Elevation	29.96	-5.75 10^{-3}		< 0.001
	MAP	-40.07	4.52 10^{-2}	-8.77 10^{-6}	< 0.001
	MAT	2.23	1.14		< 0.001
	Soil C:N ratio	33.18	-1.06		< 0.05
	pH (KCL)	177.54	-73.69	8.16	n.s.
	AGB	5.54	4.98 10^{-2}	-4.32 10^{-5}	< 0.001
	Stem density	21.01	-1.27 10^{-2}	2.19 10^{-6}	< 0.001
	Basal area	5.62	0.21		< 0.001

Table S7. Results of correlation analyses between elevation, climatic, edaphic and stand structural variables for the six communities on Mt. Kilimanjaro. Pearson correlation coefficient *r* and *P* are given. Elevation in m a.s.l., mean annual temperature in °C, mean annual precipitation in mm, aboveground biomass in Mg ha^{-1}, stem density in n ha^{-1} and basal area in m^2 ha^{-1}.

	Elevation		Mean annual precipitation		Mean annual temperature		Soil C:N ratio		pH		Aboveground biomass		Stem density	
	r	*P*	*r*	*P*	*r*	*P*	*r*	*P*	*r*	*P*	*r*	*P*	*r*	*P*
MAP	- **0.80**	**<0.001**												
MAT	- **0.98**	**<0.001**	+ **0.76**	**<0.001**										
Soil C:N ratio	+ **0.75**	**<0.001**	- 0.37	n.s.	**-** **0.74**	**<0.001**								
pH	+ 0.05	n.s.	- 0.30	n.s.	+ 0.03	n.s.	- 0.47	<0.1						
AGB	- **0.73**	**<0.001**	+ **0.66**	**<0.01**	+ **0.66**	**<0.01**	- **0.65**	**<0.01**	+ 0.00	n.s.				
Stem density	+ **0.52**	**<0.05**	- **0.51**	**<0.05**	- 0.44	n.s.	+ 0.33	n.s.	+ 0.10	n.s.	**-** **0.50**	**<0.05**		
Basal area	- **0.71**	**<0.001**	+ **0.76**	**<0.001**	+ **0.63**	**<0.01**	- **0.46**	**<0.05**	- 0.15	n.s.	+ **0.87**	**<0.001**	**-** **0.53**	**<0.05**

Climatic data from Appelhans and others (2015), topographic and stand structure data from Hemp (unpublished data), aboveground biomass data from Ensslin and others (2015) and David Schellenberger-Costa (unpublished data), soil data from Becker (unpublished data). Values in bold indicate significant correlation ($p < 0.05$). Positive correlations are marked with (+) and negative ones with (-).

Table S8. Simple linear and nonlinear regression models on the relation between fine root lifespan (log-transformed) and various root morphological and chemical traits.

		Root N content			Mean root diameter			RTD			SRA		SRL		Fine root biomass	
		r^2_{adj}/r^2	*P*		r^2_{adj}/r^2	*P*		r^2	*P*		r^2	*P*	r^2	*P*	r^2	*P*
Log (fine root lifespan)	**-**	**0.63**	**<0.001**	-	0.22	<0.1	*	0.28	<0.1	-	0.18	n.s.	$1.27\ 10^{-6}$	n.s.	**0.38**	**<0.05**
SRL	-	0.17	<0.1	**-**	**0.51**	**<0.001**	*****	0.06	n.s.		**0.40**	**<0.01**				
SRA		0.03	n.s.	*	0.06	n.s.	* **-**	**0.32**	**<0.05**							
RTD	**-**	**0.42**	**<0.01**	**-**	**0.58**	**<0.001**										
Mean root diameter		**0.29**	**<0.05**													

Mean root lifespan in years, SRL= specific root length in m g^{-1}, SRA= specific root area in $cm^2\ g^{-1}$, RTD= root tissue density in g cm^{-3}, mean diameter in mm, and root nitrogen content in mg g^{-1}, fine root biomass in Mg ha^{-1}. Significant relations are marked in bold ($P<0.05$), 2nd order polynomial relations are indicated by (*) and negative relations by (-).

Table S9. Estimates of the simple linear and non-linear regression models among fine root morphological and chemical traits and fine root lifespan along the tropical montane forest at Mt. Kilimanjaro.

Fine root variable	Factor	Intercept	Estimate (X)	Estimate (X^2)	P- value
Log (fine root lifespan)	Root N content	2.16	-0.09		< 0.001
	Mean root diameter	4.26	-10.30	7.02	< 0.1
	Root tissue density	0.14	1.63		< 0.1
	Specific root area	1.89	$-5.59\ 10^{-3}$		n.s.
	Specific root length	0.84	$1.59\ 10^{-4}$		< 0.1
Specific root length	Root N content	17.98	-0.41		< 0.1
	Mean root diameter	18.73	-2.69	-10.86	< 0.001
	Root tissue density	9.49	6.39		n.s.
	Specific root area	-0.87	0.06		< 0.01
Specific root area	Root N content	50.43	23.49	-0.82	n.s.
	Mean root diameter	8.35	626.43	-465.59	n.s.
	Root tissue density	259.25	-150.80		< 0.05
Root tissue density	Root N content	0.74	-0.02		< 0.01
	Mean root diameter	0.83	-0.67		< 0.001
Mean root diameter	Root N content	0.99	-0.86		< 0.05
